# Mendelian randomization study highlights hypothyroidism as a causal determinant of alopecia areata

**DOI:** 10.3389/fendo.2023.1309620

**Published:** 2024-01-16

**Authors:** Xue-Yong Zheng, Yan-Ping Ma, Bo Zhang, Yan-Xin Chen, Lei Tang, Xiao-Hua Tai, Jia-Hao Cao

**Affiliations:** ^1^ Department of Thyroid Surgery, The Second Affiliated Hospital of Jiaxing University, Jiaxing, China; ^2^ Department of Endoscopic Center, The Second Affiliated Hospital of Jiaxing University, Jiaxing, China

**Keywords:** alopecia areata, hypothyroidism, two-sample Mendelian randomization, SNPs, GWAS summary statistics

## Abstract

**Background:**

Although observational studies have found an association between hypothyroidism and alopecia areata, the causality of this relationship remains unclear.

**Objectives:**

This study aimed to investigate the genetic variants associated with hypothyroidism and their potential impact on the risk of developing alopecia areata.

**Methods:**

genome-wide association study summary statistics for hypothyroidism (30,155 cases and 379,986 controls) and alopecia areata (289 cases and 211,139 controls) were obtained from the IEU OpenGwas project. The inverse variance-weighted method was used as the primary analysis method to evaluate the causality between hypothyroidism and alopecia areata, supplemented by the weighted median, MR-Egger, simple mode and weighted mode. Furthermore, the function of causal SNPs was evaluated by gene ontology (GO) analysis, Kyoto Encyclopedia of Genes and Genomes (KEGG) pathway enrichment analysis, and protein–protein interaction networks.

**Result:**

Utilizing two-sample Mendelian randomization analysis, we found that the single-nucleotide polymorphisms (SNPs) of hypothyroidism (OR = 1.40, 95% CI: 1.12–1.75, *p* = 3.03×10^−3^) significantly increased the risk of alopecia areata ( 289 cases and 211,139 controls ). KEGG pathway analysis showed that the candidate genes were mainly enriched in virion-herpesvirus, Th1 and Th2 cell differentiation, Th17 cell differentiation, T-cell receptor signaling pathway, PD-L1/PD-1 checkpoint pathway in cancer and Toll-like receptor signaling pathway. Protein–protein interaction networks results showed that CTLA4, STAT4, IL2RA, TYK2, IRF7, SH2B3, BACH2, TLR3, NOD2, and FLT3.

**Conclusion:**

This study provided compelling genetic evidence supporting a causative association between hypothyroidism and alopecia areata, which could potentially inform the development of more efficacious treatment strategies for patients afflicted by alopecia areata.

## Introduction

Alopecia areata is a form of non-scarring alopecia characterized by chronic inflammation at the hair follicle level (1). The most common clinical manifestation of alopecia areata is patchy hair loss, which can progress to diffuse or complete scalp and body hair loss (2). As the second most common cause of hair loss after androgenetic alopecia, it affects approximately 2% of the global population and significantly impacts an individual’s quality of life (3). Hypothyroidism refers to a condition caused by insufficient synthesis, secretion, or biological effects of thyroid hormones (4), affecting approximately 10% of the global population (5, 6). Previous observational studies have reported an association between hypothyroidism and alopecia areata (7); however, these studies may be limited by potential confounding factors and reverse causality. To overcome these limitations, Mendelian randomization studies have gained popularity as a robust method for assessing causal relationships with reduced bias due to randomly assigned genetic variants unaffected by outcome or confounders (8, 9). By employing genetic variants as instrumental variables in this study, we aim to evaluate the potential causal effect between hypothyroidism and alopecia areata and the function of causal SNPs, which may provide valuable insights into the treatment of patients with alopecia areata.

## Methods

### Study design

**Figure 1:**
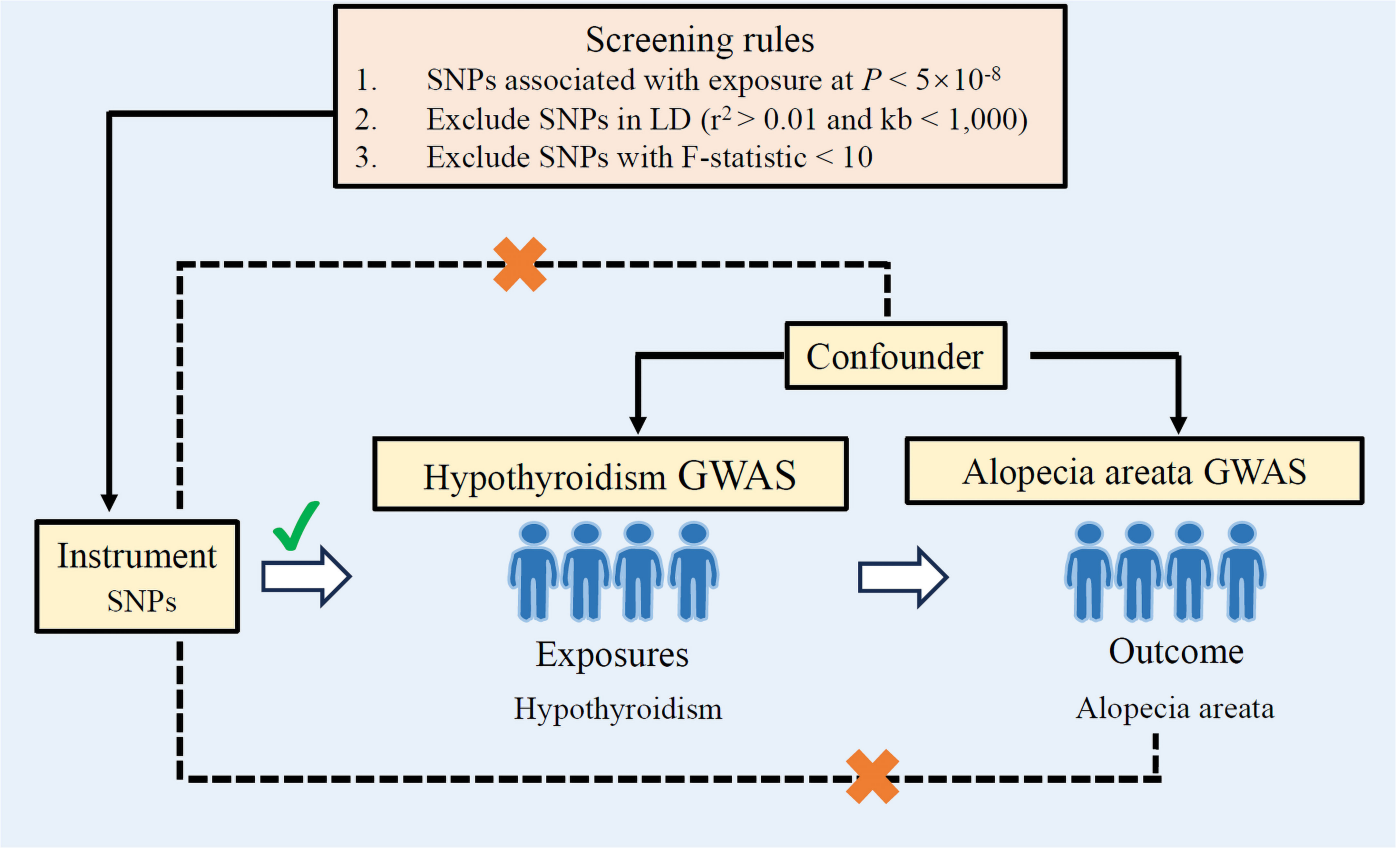
Study design overview. If genetic variants can be used as instrumental variables, the following three assumptions need to be met: (1) Genetic variants used as instrumental variables are not associated with confounders. (2) Genetic variants used as an instrumental variable is strongly associated with exposure (hypothyroidism). (3) Genetic variants used as an instrumental variable affect the outcome (alopecia areata) only through the pathway of exposure (hypothyroidism), and there are no other mediators for the effect of the genetic variant on the outcome. LD, linkage disequilibrium; GWAS, genome-wide association studies; SNPs, single-nucleotide polymorphisms.

The analytic flow of this study is illustrated in **Figure 1**. Two-sample Mendelian randomization was employed to examine the causal relationship between hypothyroidism and alopecia areata. Genome-wide association study (GWAS) summary statistics data for exposure (hypothyroidism, ebi-a-GCST90018862) and outcome (alopecia areata, finn-b-L12_ALOPECAREATA) were obtained from the IEU OpenGwas project (accessed through https://gwas.mrcieu.ac.uk/) in October 2021, which derives its original data from the FinnGen study (accessed through https://www.finngen.fi/en/access_results). The FinnGen study was designed to collect and analyze genomic information from more than 500,000 participants from the Finnish Biobank and combine it with information from national healthcare registries (10). The mean age of hypothyroidism sample was approximately 51.8 years old, and 56.3% were women. Diagnostic criteria for hypothyroidism were defined by International Classification of Diseases, 10th Revision (ICD-10), and the GWAS data for hypothyroidism contained 24,138,872 variant loci from 30,155 cases and 379,986 controls. Neuro-comorbidities were excluded in controls. Diagnostic criteria for alopecia areata are defined by ICD-8, ICD-9, and ICD-10, and GWAS statistics for alopecia areata contain 16,380,450 variant loci from 289 cases and 211,139 controls. The median age of the alopecia areata sample at first event was approximately 43.58 years old, and female patients accounted for approximately 77.86%. Disorders of skin appendages were excluded in controls. All studies were conducted in populations of European origin. More details of the data for the populations studied for hypothyroidism and pemphigus can be found at the database web site (https://risteys.finregistry.fi/). All studies included in the referenced GWAS and consortia have received approval from relevant review boards, and informed consent has been obtained from the participants involved. This study is based on publicly available summary statistics, and no ethical approval is required.

### Selection of instrumental variables

The exposure and outcome information used in this study is shown in **Table 1**. For screening of instrumental variables, a genome-wide significance threshold (1 × 10^−8^) was set to identify instrumental variables exhibiting significant correlations with exposure. Linkage disequilibrium among the instrumental variables were removed by setting the clump parameter (*r* ^2 =^ 0.01 and kb = 1,000). During reconciliation of exposure and outcome statistics, palindromes and incompatible SNPs were excluded; additionally, SNPs associated with exposures that could not be matched in the GWAS outcome statistics were also excluded. In order to mitigate potential biases stemming from weak instruments during causal analyses, SNPs with an *F*-statistic < 10 were eliminated.

**Table 1:** Data sources and information on exposure and outcome.

Exposure or outcome	Population	Sex	No. of cases	No. of controls	No. of SNPs	GWAS ID	Years
**Hypothyroidism**	European	Male and Female	30,155	379,986	24,138,872	ebi-a-GCST90018862	2021
**Alopecia areata**	European	Male and Female	289	211,139	16,380,450	finn-b-L12_ALOPECAREATA	2021

### Enrichment analysis of SNP-related genes

Gene names were included for each rsID labelled in the National Human Genome Research Institute’s Single Nucleotide Polymorphism Database (https://www.ncbi.nlm.nih.gov/snp/). The specific and common parts between the three sets of data were analyzed, and the results were visualized using R packages: ggplot2 [3.3.6]. The Database for Annotation, Visualization and Integrated Discovery (https://david.ncifcrf.gov/) is used in the GO analysis and KEGG pathway enrichment analysis. Protein–protein interaction networks were analyzed by STRING database (https://cn.string-db.org/).

### Statistical analysis

Inverse variance weighting method is the primary approach for assessing causality in two-sample Mendelian randomization analysis. Sensitivity analysis mainly included heterogeneity test, horizontal pleiotropy test, and leave-one-out analysis. It should be noted that instrumental variables from different platforms, experiments, populations, etc., may exhibit heterogeneity, which can ultimately impact the results. Inverse variance weighted and MR Egger are essentially meta-analyses, and Cochrane Q can be obtained by testing data for heterogeneity. A *p*-value of less than 0.05 was considered statistically significant for indicating heterogeneity among the included SNPs. The horizontal pleiotropy test was used to determine whether confounding factors were present in the study. The MR-Egger intercept test and MR-PRESSO test were used for horizontal pleiotropy test, and a *p-*value less than 0.05 was considered statistically significant, indicating evidence of horizontal pleiotropy among the included SNPs. Leave-one-out analysis removes each SNP step by step, calculates the meta effect of the remaining SNPs, and determines whether the removal of a SNP causes significant changes to the results. After removing each SNP, the total error line changes little, indicating that the results are reliable. Statistical analyses were conducted using TwoSampleMR, MR-PRESSO, and MendelianRandomization packages within R software (version 4.3).

## Results

### Hypothyroidism significantly associated with increased risk of alopecia areata

**Figure 2:**
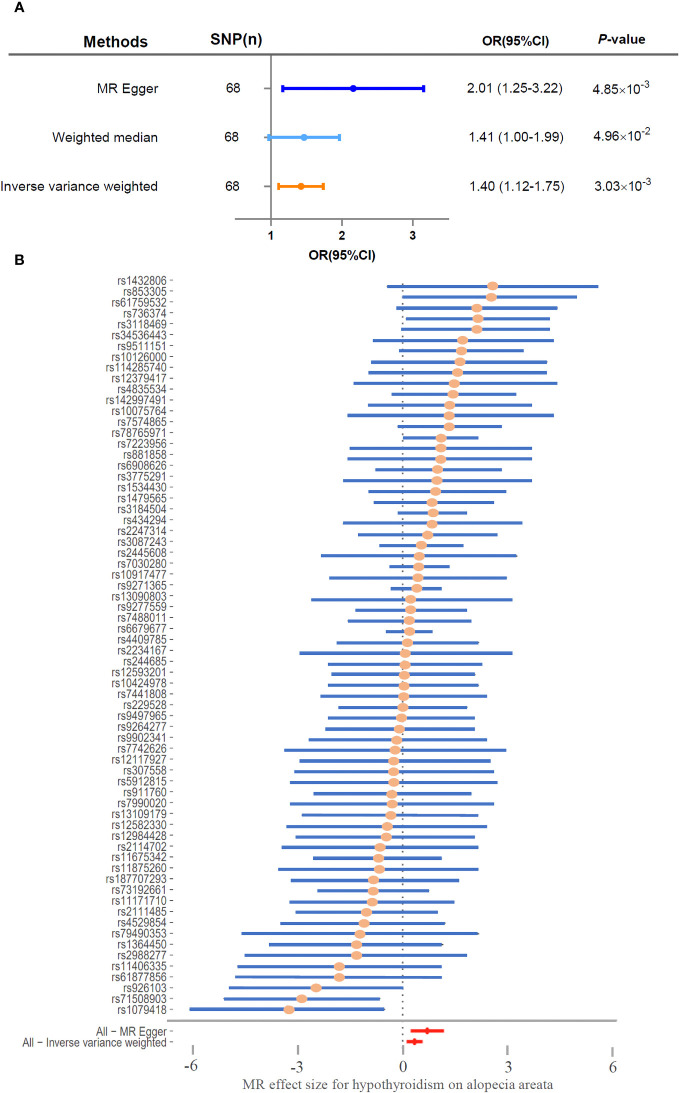
Hypothyroidism significantly associated with risk of alopecia areata. **(A)** Two-sample Mendelian randomization analyzed the risk of hypothyroidism and alopecia areata. **(B)** Forest plot of MR effect size for hypothyroidism on alopecia areata. SNP, single-nucleotide polymorphism; CI, confidence interval; OR, odds ratio.

The genetic SNPs used in the two-sample Mendelian randomization analysis between hypothyroidism and alopecia areata were selected based on locus-wide significance (locus-wide significance, *p* < 1 × 10^-8^) (**Supplementary Table 1**). The following SNPs were removed for being palindromic with intermediate allele frequencies: rs2412976 and rs2921053. After screening, a total of 68 SNPs met the inclusion criteria (**Figure 1**). MR Egger (OR = 2.01, 95% CI: 1.25–3.22, *p* = 4.85×10^−3^), weighted median (OR = 1.41, 95% CI: 1.00–1.99, *p* = 4.96×10^−2^), and inverse variance weighting method (OR = 1.40, 95% CI: 1.12–1.75, *p* = 3.03×10^−3^) were utilized for Mendelian randomization analysis; these analyses consistently demonstrated that the SNPs of hypothyroidism significantly increased the risk of alopecia areata (**Figure 2A**). To ensure robustness and reliability of our findings, two methods, namely, inverse variance weighted analysis (*p* = 0.38) and MR-Egger analysis (*p* = 0.44), were utilized for heterogeneity testing (**Table 2**). Furthermore, to assess potential confounding factors in this study, the MR-Egger intercept test (*p* = 0.09) and MR-PRESSO test (*p* = 0.42) were utilized for horizontal pleiotropy. The results showed that the difference was not statistically significant, indicating the absence of horizontal pleiotropy (**Table 2**). Additionally, leave‐one‐out analyses showed no single SNP driving the results (**Supplementary Figure 1**). As shown in **Figure 2B**, the forest plot displayed the effect value of each SNP. A total of 40 SNPs related to hypothyroidism were found to be positively associated with alopecia areata. It is worth noting that rs1432806, rs853305, rs1759532, rs736374, and rs3118469 in hypothyroidism may significantly contribute to the incidence of alopecia areata. The MR effect size of All-MR Egger (*b* = 0.70) and All-Inverse variance weighted (*b* = 0.33) displayed that hypothyroidism could increase the risk of alopecia areata (**Figure 2B**).

**Table 2:** Sensitivity test of Mendelian randomization analyze of the associations between hypothyroidism and risk of alopecia areata.

Exposures	Heterogeneity test						Horizontal pleiotropy test				
	Inverse variance weighted			MR-Egger			MR-Egger intercept test		MR-PRESSO test		
	*Q*	*Q*_df	*p*	*Q*	Q_df	*p*	SE	*p*	Outlier corrected	Outlier snp	*p*
Hypothyroidism	69.87	67	0.38	66.91	66	0.44	0.02	0.09	NA	NA	0.42

NA, Not Available. NA is considered to have no outliers detected.

### Functional evaluation of causal SNPs

**Figure 3:**
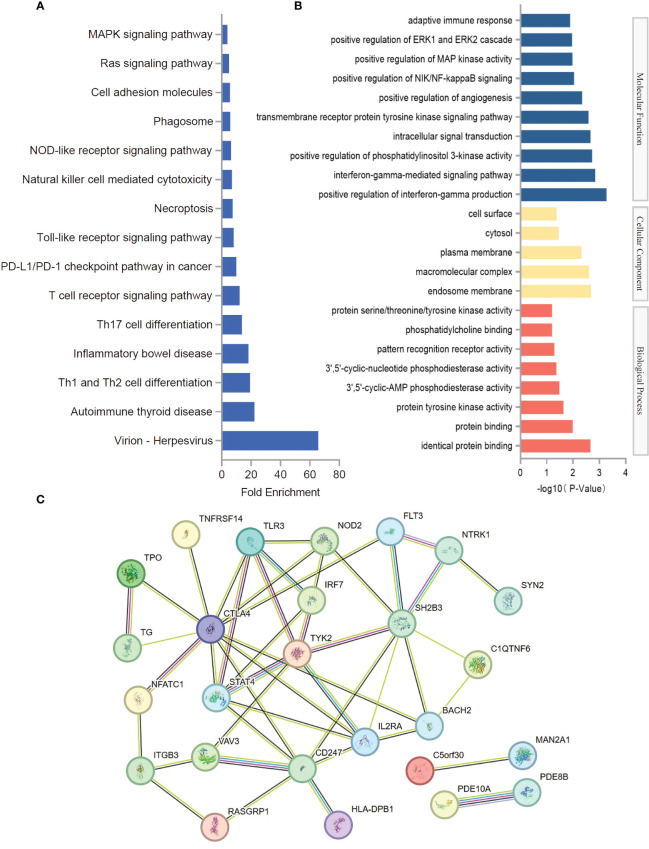
Functional evaluation of causal SNPs. **(A)** KEGG pathway analysis of SNP-related genes. **(B)** GO enrichment analysis of SNP-related genes. **(C)** Protein–protein interaction networks of SNP-related genes.

In order to further understand the function of causal SNPs, candidate genes corresponding to each rsID were analyzed (**Supplementary Table 2**). To provide comprehensive insights into the biological processes associated with these genes, GO enrichment analysis and KEGG pathway analysis were conducted. These analyses integrate functional annotations from various genes, allowing for predictions on the specific biological processes linked to different gene sets. KEGG pathway analysis showed that the candidate genes were mainly enriched in virion-herpesvirus, Th1 and Th2 cell differentiation, inflammatory bowel disease, Th17 cell differentiation, T-cell receptor signaling pathway, PD-L1/PD-1 checkpoint pathway in cancer, Toll-like receptor signaling pathway, necroptosis, natural killer cell mediated cytotoxicity, and NOD-like receptor signaling pathway (**Figure 3A**) (**Supplementary Table 3**). GO enrichment analysis showed that the candidate genes were mainly enriched in positive regulation of interferon-gamma production, interferon-gamma-mediated signaling pathway, positive regulation of phosphatidylinositol 3-kinase activity, intracellular signal transduction, endosome membrane, macromolecular complex, plasma membrane, identical protein binding, protein binding, and protein tyrosine kinase activity (**Figure 3B**, **Supplementary Table 3**). Protein–protein interaction networks results showed that CTLA4, STAT4, IL2RA, TYK2, IRF7, SH2B3, BACH2, TLR3, NOD2, and FLT3 may play a role in the association mechanism between hypothyroidism and stroke (**Figure 3C**).

## Discussion

Previous studies have indicated that hypothyroidism was associated with a higher risk of alopecia areata (11, 12). Specifically, in a cohort of 89 individuals with alopecia areata (median age: 40 years; male–female ratio: 0.17), a concurrent diagnosis of hypothyroidism was reported (13). Similarly, another study involved 78 newly diagnosed alopecia areata patients (male–female ratio: 1.44; mean age: 32.7 ± 3.09 years) attending a community dermatology clinic between 2007 and 2011. Of these, 13 patients (16.6%) had hypothyroidism (14). Additionally, a prospective study utilizing data from the Nurses’ Health Study, which included 63,692 women aged 53–80 years, demonstrated that a history of hypothyroidism was associated with an increased risk of alopecia areata (HR 1.88, 95% CI 1.30–2.71) (15). Building upon these findings, our study employed a two-sample Mendelian randomization design to investigate the causal relationship between hypothyroidism and alopecia areata. Our analysis supported a causal association between these two conditions. The study did not assess the association between the degree of thyroid hormone reduction and the severity of alopecia areata. However, the correlation between the degree of thyroid hormone reduction and the severity of alopecia areata was not assessed in this study. A systematic meta-analysis showed that patients with alopecia areata were more likely to have thyroid dysfunction (OR = 4.36; 95% CI 1.19–5.99; prevalence of 12.5%), especially subclinical hyperthyroidism (OR = 5.55; 95% CI 1.73–7.85; prevalence of 5.7%) and subclinical hypothyroidism (OR = 19.61; 95% CI 4.07–94.41; prevalence of 10.4%) (16). Abnormalities in both thyroid hormones and antithyroid antibodies are likely to increase the risk of alopecia areata, but the correlation between thyroid hormones and antithyroid antibodies and the severity of alopecia areata still need to be elucidated in more studies.

The biological connection between hypothyroidism and alopecia areata has not yet been fully elucidated. Currently, hypothyroidism has important effects on hair follicle growth and development and the immune system, which may explain the causal relationship between hypothyroidism and alopecia areata that we observed in our current study. Decreased thyroid hormone levels caused by hypothyroidism can affect the growth and development of hair follicles. Thyroid hormone plays an important role in the growth cycle of hair; it regulates the proliferation and differentiation of hair follicles (17). When thyroid hormone levels are insufficient, these biological processes may be affected, leading to thinning or loss of hair (18). In addition, hypothyroidism may also lead to an abnormal immune system response, which can lead to autoimmune alopecia areata. Autoimmune alopecia areata is a condition in which the immune system attacks the hair follicles, causing hair loss (19, 20). In patients with hypothyroidism, an abnormal immune system may increase the risk of autoimmune alopecia areata (21, 22). However, it is important to note that not all patients with hypothyroidism develop alopecia areata. This may be due to individual differences, disease severity, treatment measures, and other factors. In clinical practice, it is necessary to examine patients with alopecia areata for thyroid abnormalities and to further determine prevention and treatment methods.

Despite the valuable insights gained from our study, there are certain limitations to consider. First, owing to the limitations of SNP screening and the small sample size of alopecia areata, it is difficult to assess whether there is a reverse causality between hypothyroidism (exposure) and alopecia areata (outcome). Second, our study was limited to individuals of European descent, which not only reduces bias due to ethnic and regional differences but also limits the generalizability of our findings to other populations. Lastly, although our study provides genetic evidence for a causal relationship, additional research, including functional studies, is necessary to further elucidate the underlying mechanisms.

## Conclusion

The research provided genetic evidence supporting a causal association between hypothyroidism and alopecia areata. The hypothyroidism was associated with a higher risk of alopecia areata. The function of causal SNP-related genes was assessed by GO enrichment analysis, KEGG pathway analysis, and protein–protein interaction networks, which provided ideas for further investigation of the mechanism by which hypothyroidism increases the risk of alopecia areata. These findings contribute to a better understanding of the underlying mechanisms and may guide future prevention and treatment strategies. Implementing routine screening for thyroid hormone may help optimize treatment strategies for alopecia areata and may lead to better clinical outcomes for patients with alopecia areata.

## Data availability statement

The original contributions presented in the study are included in the article/**Supplementary Material**. Further inquiries can be directed to the corresponding authors.

## Author contributions

X-YZ: Data curation, Writing – review & editing, Project administration. Y-PM: Conceptualization, Writing – original draft. BZ: Methodology, Writing – original draft. Y-XC: Investigation, Supervision, Writing – review & editing. LT: Conceptualization, Data curation, Writing – review & editing. X-HT: Data curation, Methodology, Writing – original draft. J-HC: Conceptualization, Data curation, Writing – original draft, Writing – review & editing.

## Supplementary material

Supplementary Figure 1 No single SNP driving the results in leave‐one‐out analyses.

Supplementary Table 1 Characteristics of the genetic SNPs used in two-sample Mendelian randomization analysis between hypothyroidism and alopecia areata.

Supplementary Table 2 Candidate genes corresponding to each rsID in the causal SNPs.

Supplementary Table 3 Gene ontology analysis and Kyoto Encyclopedia of Genes and Genomes pathway enrichment analysis of SNPs related genes.
