# Supplementary figures and images for: Mendelian randomization study highlights hypothyroidism as a causal determinant of alopecia areata
Source: Front Endocrinol (Lausanne). 2024 Jan 16;14:1309620. doi: 10.3389/fendo.2023.1309620 (PMC10826415; doi:10.3389/fendo.2023.1309620)

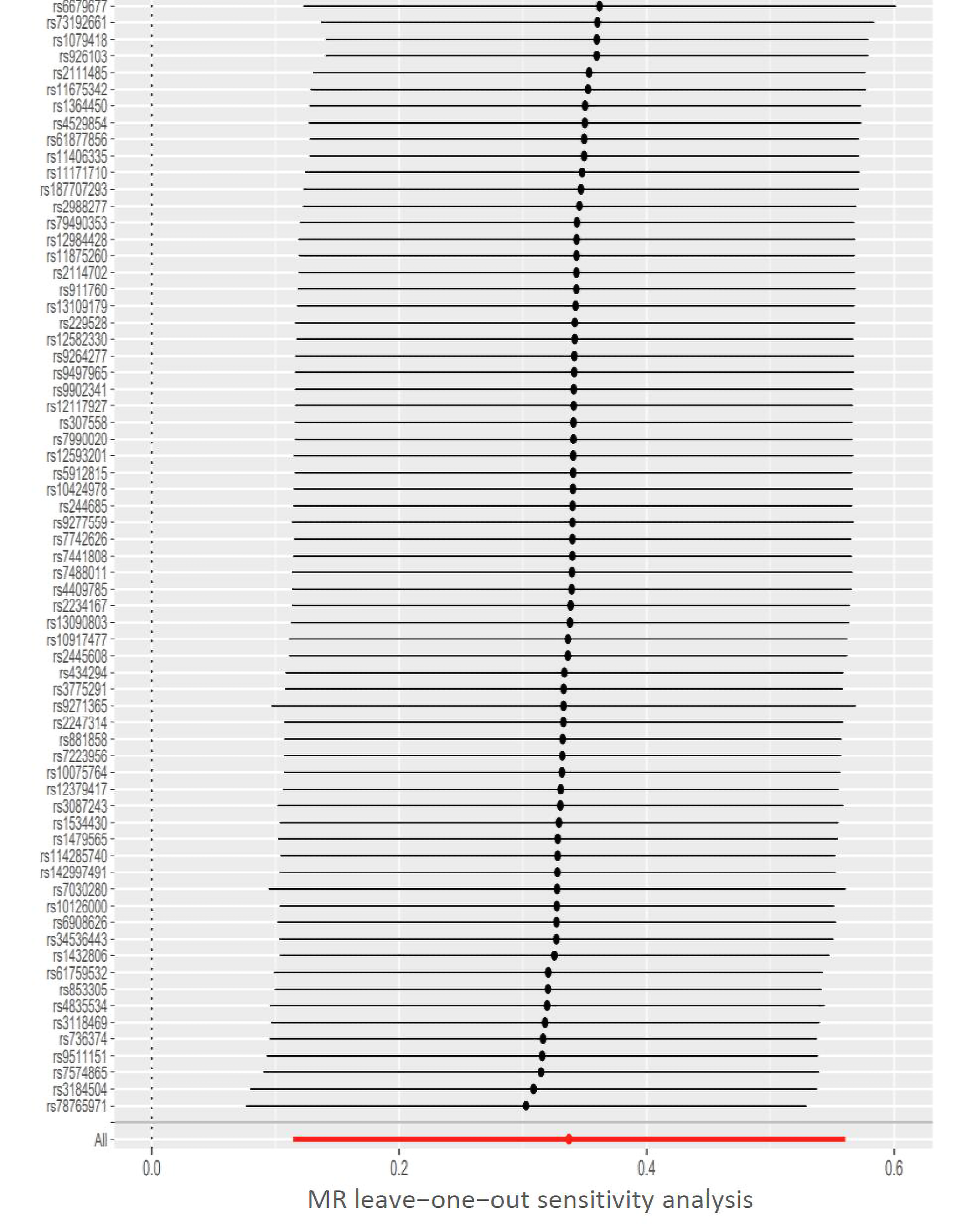

Supplement: Supplementary Figure 1 — No single SNP driving the results in leave‐one‐out analyses. [file Image_1.tif]
